# Análise tecnológica e de mercado do segmento de membranas de hemodiálise no Brasil com base nos dados de compras públicas federais

**DOI:** 10.1590/0102-311XPT106625

**Published:** 2026-06-26

**Authors:** Gustavo Feliciano de Jesus Barcelos, Beatriz Godoi de Nogueira, Kátia Cecília de Souza Figueiredo, Carlos Alberto Tagliati

**Affiliations:** 1 Programa de Pós-graduação em Inovação Tecnológica, Universidade Federal de Minas Gerais, Belo Horizonte, Brasil.; 2 Programa de Pós-graduação em Engenharia Química, Universidade Federal de Minas Gerais, Belo Horizonte, Brasil.

**Keywords:** Unidades Hospitalares de Hemodiálise, Diálise Renal, Administração em Saúde, Hospital Hemodialysis Units, Renal Dialysis, Health Administration, Unidades de Hemodiálisis en Hospital, Diálisis Renal, Administración en Salud

## Abstract

Este estudo analisa o mercado e a tecnologia do segmento de membranas de hemodiálise no Brasil, com base nas aquisições públicas realizadas pela Empresa Brasileira de Serviços Hospitalares (EBSERH). A insuficiência renal, que afeta cerca de 10% da população mundial, representa um desafio crescente para os sistemas de saúde, sendo o Brasil altamente dependente de importações para suprir a demanda por dialisadores. Entre agosto de 2022 e julho de 2023, a EBSERH adquiriu 144.162 unidades de dialisadores, provenientes de seis fabricantes internacionais, evidenciando a ausência de produção nacional e uma grande dependência tecnológica do país. Adicionalmente, foram avaliadas as características físicas e de transporte das membranas, como materiais utilizados (predominância de polissulfonas), dimensões e coeficiente de ultrafiltração. Os resultados apontam a necessidade de políticas públicas robustas para fomentar a produção nacional, reduzindo custos, promovendo inovação e fortalecendo a autonomia tecnológica do país. Este trabalho fornece subsídios para o planejamento estratégico do setor de hemodiálise, identificando lacunas e oportunidades para o desenvolvimento de uma indústria nacional de membranas de hemodiálise.

## Abstract (en)

This study analyzes the market and technology of the hemodialysis membrane segment in Brazil, based on public acquisitions made by the Brazilian Company of Hospital Services (EBSERH, acronym in Portuguese). Kidney failure, which affects about 10% of the world’s population, represents a growing challenge for health systems, and Brazil is highly dependent on imports to meet the demand for dialyzers. From August 2022 to July 2023, EBSERH acquired 144,162 dialyzers, from six international manufacturers, evincing the absence of a national production and a great technological dependence on Brazil. Additionally, the physical and transport characteristics of the membranes were evaluated, such as materials used (predominance of polysulfone), dimensions and ultrafiltration coefficient. The results point to the need for robust public policies to foster national production, reducing costs, promoting innovation and strengthening Brazil’s technological autonomy. This work provides relevant information for the strategic planning of the hemodialysis sector, identifying gaps and opportunities for the development of a national hemodialysis membrane industry.

## Abstract (es)

Este estudio analiza el mercado y la tecnología del segmento de membranas de hemodiálisis en Brasil, con base en las adquisiciones públicas realizadas por la Empresa Brasileña de Servicios Hospitalarios (EBSERH). La insuficiencia renal, que afecta a cerca del 10% de la población mundial, representa un desafío creciente para los sistemas de salud, y Brasil es altamente dependiente de las importaciones para satisfacer la demanda de dializadores. Entre agosto del 2022 y julio del 2023, Ebserh adquirió 144.162 unidades de dializadores de seis fabricantes internacionales, lo que pone de relieve la falta de producción nacional y una importante dependencia tecnológica del país. Además, se evaluaron las características físicas y de transporte de las membranas, como los materiales utilizados (predominantemente polisulfonas), dimensiones y coeficiente de ultrafiltración. Los resultados señalan la necesidad de políticas públicas sólidas para fomentar la producción nacional, reducir costos, promover la innovación y fortalecer la autonomía tecnológica del país. Este trabajo aporta insumos para la planificación estratégica del sector de hemodiálisis, identificando brechas y oportunidades para el desarrollo de una industria nacional de membranas de hemodiálisis.

## Introdução

A insuficiência renal é um dos principais problemas de saúde global, afetando aproximadamente 10% da população mundial ^1^. Dentro desse grupo, cerca de 3,2 milhões de pacientes necessitam de tratamento por hemodiálise, um número que cresce, anualmente, a uma taxa de 6% ^1^. 

Esse aumento da demanda representa um desafio para os sistemas de saúde, que precisam suprir a crescente necessidade por equipamentos e insumos essenciais para o tratamento dialítico. No Brasil, a situação é particularmente preocupante, uma vez que 79% procedimentos de hemodiálise são financiados pelo Sistema Único de Saúde (SUS), que lida com o impacto dos altos custos e com a dependência de importações para garantir o tratamento de milhares de brasileiros ^2^.

Segundo dados do Ministério da Saúde ^3^, o Brasil possui 723 estabelecimentos habilitados para terapias renais substitutivas, atendendo 148 mil pacientes. No entanto, a maioria dos insumos e equipamentos necessários para hemodiálise é importada, o que eleva os custos desse tratamento essencial ^4^ ^,^ ^5^.

O tratamento da insuficiência renal crônica foi introduzido no Brasil na década de 1970 com o uso de hemodialisadores importados, principalmente da empresa norte-americana Travenol, atualmente conhecida como Baxter. Com o crescimento do serviço de hemodiálise no país, surgiram algumas fabricantes nacionais, como Macchi e Sistemas Vitais, para fornecer insumos e equipamentos ^6^. Entretanto, na década de 1990, o mercado nacional enfrentou retração significativa devido ao impacto de eventos como o acidente de Caruaru (Pernambuco), somado ao aumento das regulamentações e à forte concorrência internacional. 

Atualmente, o setor é amplamente dominado por empresas estrangeiras, como Fresenius, Nipro e Baxter, o que evidencia uma dependência externa no suprimento de dialisadores e outros insumos críticos ^7^ ^,^ ^8^. De acordo com dados obtidos no Comex Stat (http://comexstat.mdic.gov.br/pt/home), portal do governo brasileiro para acesso gratuito às estatísticas de comércio exterior no Brasil, no ano de 2022 as importações de insumos para hemodiálise atingiram um valor cerca de 120 vezes superior às exportações, totalizando quase 75 milhões de dólares ^2^.

Esse problema não é exclusivo do setor de dialisadores, ele abrange diversos segmentos da saúde, como as indústrias de vacinas e de ingredientes farmacêuticos ativos. A produção nacional de vacinas, por exemplo, é exclusivamente pública e essencialmente dependente da transferência de tecnologia estrangeira ^9^. A fabricação interna de ingredientes farmacêuticos ativos, por sua vez, corresponde a apenas 5% da demanda das empresas farmacêuticas brasileiras ^10^.

A Lei da Inovação ^11^, de 2004, e outras políticas públicas ressaltam o papel do Estado em utilizar seu poder de compra para incentivar a inovação e a capacitação tecnológica no Brasil. No setor de hemodiálise, a aplicação desse princípio, por meio de compras públicas direcionadas, pode reduzir a dependência de importações, diminuir custos, gerar empregos e promover o desenvolvimento de novas tecnologias locais ^2^.

O Programa Nova Indústria Brasil ^12^, lançado em 2024, reforça essa estratégia ao destinar 300 bilhões de Reais para fomentar setores estratégicos, incluindo a saúde. O objetivo do programa é que, até 2033, o Brasil seja capaz de atender 70% de suas necessidades em medicamentos, vacinas, dispositivos médicos e tecnologias de saúde, fortalecendo a produção nacional e reduzindo a dependência externa. Para que essas políticas sejam eficazes, é essencial contar com uma base sólida de dados e indicadores, que orientem os investimentos e identifiquem oportunidades de crescimento.

A dependência do Brasil em importações no setor de hemodiálise representa uma importante lacuna no desenvolvimento tecnológico da saúde no país. Reduzir essa vulnerabilidade exige uma compreensão aprofundada da dinâmica do mercado e a identificação de oportunidades para desenvolver tecnologias nacionais. Uma política tecnológica robusta deve estar fundamentada em indicadores precisos, que permitam implementar medidas de fomento à inovação local.

Nesse contexto, este estudo tem como objetivo realizar uma análise tecnológica e de mercado das membranas de hemodiálise no Brasil, investigando o panorama atual e identificando oportunidades para o desenvolvimento de uma indústria nacional nesse setor. A análise dos principais fabricantes e das importações associadas às membranas fornecerá dados estratégicos que deixarão clara a necessidade de formulação de políticas públicas e de incentivo à inovação local na área. 

Embora, conforme citado anteriormente, o problema da importação de produtos estrangeiros em detrimento da produção nacional não seja uma novidade, a originalidade deste trabalho consiste no enfoque específico dado ao segmento de hemodiálise. Além disso, não há relatos de estudos na literatura que tenham realizado o mesmo tipo de análise de dados de compra de dialisadores pela Empresa Brasileira de Serviços Hospitalares (EBSERH), destacando as empresas fornecedoras e as características dos produtos fornecidos.

## Metodologia

A metodologia adotada neste estudo teve como objetivo mapear as características das membranas de hemodiálise comercializadas no Brasil, dada a relevância estratégica desse insumo para o setor de saúde. Para isso, foi conduzida uma análise dos dados de licitações públicas, com foco em identificar o perfil de aquisição, a origem dos produtos e os principais fornecedores envolvidos.

Dado que o SUS é o principal consumidor desse produto e seus dados de compras são disponibilizados publicamente para garantir transparência, foram selecionadas para análise as aquisições públicas de membranas de hemodiálise do SUS. Optou-se por focar nas compras públicas federais, pois, ao contrário do que acontece em estados e municípios, na esfera federal as compras são realizadas de modo centralizado. Para garantir critérios e organização padronizados, foram examinadas especificamente as transações realizadas pela EBSERH ^13^.

Os dados de compras da EBSERH estão disponíveis no Portal de Compras do Governo Federal ^13^, uma plataforma integrada que permite o monitoramento e o controle das etapas de compras e contratações públicas. Neste trabalho, foram consultados os dados do Catálogo Unificado de Materiais (CATMAT) e do Painel de Preços. 

O CATMAT é uma base de dados que classifica todos os bens licitados e serviços contratados pela Administração Pública Federal, atribuindo códigos específicos para cada item, o que evita problemas de ambiguidade e permite consultas em outros módulos do portal ^14^. A busca pelo termo “dialisador” no CATMAT resultou em vinte códigos relevantes: 454363, 454364, 454367, 454368, 454369, 454370, 454371, 454372, 454373, 454374, 454375, 454376, 454377, 454378, 454379, 454380, 454381, 454382, 454383 e 454384. 

O Painel de Preços, por sua vez, é uma base de dados onde são registrados todas as compras homologadas, incluindo documentos como editais e atas, com informações detalhadas sobre valores, quantidades adquiridas e fornecedores ^14^. Assim, foram pesquisadas no Painel de Preços todas as aquisições realizadas pela EBSERH entre 1º de agosto de 2022 e 31 de julho de 2023, para materiais classificados nos vinte códigos CATMAT identificados. 

O período investigado está alinhado aos intervalos de tempo comumente avaliados em estudos sobre processos licitatórios, que usualmente pesquisam licitações realizadas ao longo de um ou dois anos ^15^ ^,^ ^16^ ^,^ ^17^. Embora 12 meses seja um período curto para sustentar tendências de mercado ou implicações estruturais, a ampliação da coleta de dados para períodos anteriores a agosto de 2022 resultaria na aquisição de dados referentes ao período da pandemia de COVID-19, o que, naturalmente, traria para o estudo incertezas inerentes à influência desse período atípico nos padrões observados.

Todos os processos encontrados na busca foram analisados, não sendo aplicado qualquer tipo de critério de exclusão além dos já citados no parágrafo anterior. Durante essa análise foram extraídos dados como o hospital solicitante, a distribuidora vencedora da licitação, o preço e a quantidade de dialisadores adquiridos, bem como o fabricante e modelo dos produtos fornecidos. Devido à padronização do banco de dados do Painel de Preços e dos próprios processos licitatórios da EBSERH, não houve nenhum tipo de inconsistência entre os dados que precisasse ser sanada.

Para cada modelo de dialisador encontrado, foi realizada uma consulta aos catálogos dos fabricantes para obter informações específicas, como material, área de superfície, coeficiente de ultrafiltração e depuração. Além disso, foram coletados dados sobre os fabricantes, incluindo localização, número de funcionários e perfil de atuação no Brasil, a partir de fontes institucionais e documentos oficiais, como relatórios anuais.

## Resultados e discussão

### Fabricantes dos dialisadores adquiridos pela EBSERH

Entre agosto de 2022 e julho de 2023, a EBSERH realizou 31 licitações para a compra de dialisadores, totalizando 144.162 unidades adquiridas, correspondendo a 1,8% do total importado pelo Brasil nesse período. Considerando a diversidade de hospitais geridos pela instituição em todas as regiões do país e o nível de detalhamento necessário para esta pesquisa, esses processos foram considerados representativos do mercado brasileiro de membranas de hemodiálise.

A utilização de amostras dessa ordem de magnitude encontra respaldo, por exemplo, em diretriz metodológica reconhecida internacionalmente elaborada pelo Fundo Monetário Internacional (FMI) para servir como um guia técnico para a construção, cálculo e interpretação dos Índices de Preços ao Produtor ^18^. Nesse documento, o FMI admite que, em determinadas circunstâncias, como em estratos compostos por numerosos estabelecimentos de pequeno porte, pode ser considerada aceitável a aplicação de taxas de amostragem em torno de 2%, desde que garantida a representatividade da amostra ^18^.

Além disso, embora o percentual de 1,8% possa ser considerado baixo, a disponibilidade de dados a respeito da compra de dialisadores que possam ser comparados e avaliados em conjunto é de difícil obtenção. Isso ocorre porque estados e municípios também realizam diretamente suas compras e reportam essas compras em bancos de dados individuais e independentes, obedecendo a padrões diferentes de organização das informações. Ainda de modo comparativo, os dados do censo brasileiro de diálise de 2022 e 2023 ^19^ ^,^ ^20^ indicam que apenas 28% e 37,5% dos centros de diálise ativos, respectivamente, responderam ao questionário disponibilizado. Isso revela uma dificuldade grande de acesso a dados do número de pacientes, devendo ser levado em consideração que os dados de compras são ainda mais sensíveis, principalmente em empresas privadas. 

No Quadro 1 estão apresentadas as principais informações das 31 licitações encontradas nesta pesquisa. A análise do quadro revelou que as 31 licitações foram vencidas por produtos de apenas seis empresas, todas internacionais, demonstrando a dependência brasileira e a dominância de fabricantes estrangeiros no setor de dialisadores. Isso evidencia a importância de fomentar a indústria nacional de equipamentos e insumos para hemodiálise. Nas próximas seções será apresentada uma visão geral sobre cada uma das seis fabricantes listadas no Quadro 1.

**Quadro 1:** Compra de dialisadores pela Empresa Brasileira de Serviços Hospitalares (EBSERH) entre agosto de 2022 e julho de 2023.

FABRICANTE	DISTRIBUIDORAS	INSTITUIÇÕES DEMANDANTES DOS DIALISADORES ADQUIRIDOS	MODELOS	NÚMERO DE LICITAÇÕES VENCIDAS	NÚMERO DE DIALISADORES ADQUIRIDOS	PREÇO MÉDIO (R$)	VALOR TOTAL DAS VENDAS (R$)
Allmed	Alfa Hospitalar; Allmed; Inovamed	Complexo Hospitalar do Ceará (CE); Empresa Brasileira de Serviços Hospitalares (DF); Hospital de Clínicas do Triângulo Mineiro (MG); Hospital Universitário Alcides Carneiro (PB); Hospital Universitário Onofre Lopes (RN); Hospital Universitário Prof. Alberto Antunes (AL)	H1; H4; PS100; PS160; PS180	6	9.310	44,23	411.768
Bain	Diálise Com.; Fortecare; Intensivemed; Medcorp; Taurovita	Complexo Hospitalar Universitário da UFPA (PA); Empresa Brasileira de Serviços Hospitalares (DF); Hospital de Clínicas da UFU (MG); Hospital de Clínicas de Minas Gerais (MG); Hospital de Clínicas do Triângulo Mineiro (MG); Hospital Universitário Cassiano de Moraes (ES); Hospital Universitário de Brasília (DF); Hospital Universitário de Juiz de Fora (MG); Hospital Universitário de Santa Maria (RS); Hospital Universitário do Piauí (PI); Hospital Universitário Onofre Lopes (RN); Hospital Universitário Prof. Alberto Antunes (AL)	B-14H; B-16H; B-18H; B-20H; B-20PF	11	62.242	47,41	2.950.950
B. Braun	B. Braun	Hospital de Clínicas da UFU (MG); Hospital de Clínicas de Minas Gerais (MG); Hospital Universitário Alcides Carneiro (PB); Hospital Universitário Prof. Edgard Santos (BA)	PRO13H; PRO16L; PRO19H; PRO19L	4	5.952	54,43	324.021
Fresenius	Fresenius; Nova Médica	Complexo Hospitalar do Ceará (CE); Complexo Hospitalar Universitário da UFPR (PR); Complexo Hospitalar Universitário da UFPA (PA); Empresa Brasileira de Serviços Hospitalares (DF); Hospital de Clínicas da UFU (MG); Hospital de Clínicas de Goiás (GO); Hospital de Clínicas de Minas Gerais (MG); Hospital de Clínicas de Pernambuco (PE); Hospital de Clínicas do Triângulo Mineiro (MG); Hospital Universitário Alcides Carneiro (PB); Hospital Universitário Cassiano de Moraes (ES); Hospital Universitário de Juiz de Fora (MG); Hospital Universitário de Santa Maria (RS); Hospital Universitário Maria Pedrossian (MS); Hospital Universitário Onofre Lopes (RN); Hospital Universitário Prof. Alberto Antunes (AL); Hospital Universitário Prof. Edgard Santos (BA); Hospital Universitário Prof. Polydoro Thiago (SC)	CL. 100; CLASS 80; CLASS 60; CLASS 40; FX PAED; F40S; F50S; F60S; F70S; F80S; HDF80S; HDF100S; 600S; 1000S; HPSF7; AV1000S; EMIC2	21	37.176	149,52	5.558.557
Gambro (Baxter)	Baxter	Hospital de Clínicas de Goiás (GO); Hospital de Clínicas de Pernambuco (PE); Hospital Universitário Onofre Lopes (RN)	14L; 21L	3	8.750	47,07	411.862
Nipro	M Med; Nipro; R Core	Complexo Hospitalar do Ceará (CE); Hospital de Clínicas da UFU (MG); Hospital de Clínicas de Goiás (GO); Hospital de Clínicas de Minas Gerais (MG); Hospital de Clínicas do Triângulo Mineiro (MG); Hospital Universitário Alcides Carneiro (PB); Hospital Universitário de Juiz de Fora (MG); Hospital Universitário Onofre Lopes (RN); Hospital Universitário Prof. Alberto Antunes (AL); Hospital Universitário Prof. Edgard Santos (BA); Hospital Universitário Prof. Polydoro Thiago (SC)	15H; 15L; 17H; 17L; 19H; 19L; 21H; 21L	11	20.732	60,84	1.261.252

AL: Alagoas; BA: Bahia; CE: Ceará; DF: Distrito Federal; ES: Espírito Santo; GO: Goiás; MG: Minas Gerais; MS: Mato Grosso do Sul; PA: Pará; PB: Paraíba; PE: Pernambuco; PI: Piauí; PR: Paraná; RN: Rio Grande do Norte; RS: Rio Grande do Sul; SC: Santa Catarina; UFPA: Universidade Federal do Pará; UFPR: Universidade Federal do Paraná; UFU: Universidade Federal de Uberlândia.

#### Allmed

A Allmed é uma empresa inglesa especializada em produtos para hemodiálise, com unidades de fabricação na Alemanha, Portugal e Egito, e presença internacional por meio de instalações dedicadas ao marketing, às vendas e à distribuição no Brasil, Turquia, Polônia e Índia ^21^. Com mais de 1.300 funcionários, a empresa exporta para mais de 40 países. No Brasil, seu portfólio inclui dialisadores e linhas de sangue para hemodiálise, sendo distribuída principalmente pela Allmed Pronefro, localizada no Paraná, embora também conte com outras distribuidoras, como Alfa Hospitalar e Inovamed Hospitalar, que fornecem seus produtos à EBSERH ^21^. 

De acordo com os dados do Quadro 1, embora a fabricante tenha vencido cerca de 20% das licitações realizadas pela EBSERH, a Allmed teve uma participação significativa em licitações de menor escala, especialmente na Região Nordeste, com preços inferiores aos médios pagos pela instituição.

#### Bain Medical

A Bain Medical, fabricante chinesa de equipamentos médicos para hemodiálise, foi fundada em 2003 com um capital registrado superior a R$ 400 milhões. A empresa possui sete fábricas, sendo cinco na China e duas na Malásia, além de subsidiárias em Hong Kong, Taiwan, Turquia, Paquistão e Índia ^22^. Com aproximadamente 5 mil funcionários em todo o mundo, a Bain é a maior fabricante de equipamentos médicos consumíveis para hemodiálise, comercializando seus produtos em mais de 80 países ^22^. No Brasil, a empresa é representada por um escritório em São Paulo, mas não mantém distribuidora própria, optando por parceiros independentes ^22^. 

Estão apresentadas no Quadro 1 as principais informações sobre as aquisições de dialisadores da Bain Medical pela EBSERH. De acordo com o quadro, os dialisadores da Bain foram os mais adquiridos pela EBSERH no período analisado, representando 42,9% do total comprado, com presença em todas as regiões do país. Essa alta participação deve-se principalmente ao preço médio de seus produtos, que foi significativamente inferior ao preço médio dos dialisadores adquiridos pela EBSERH (R$ 75,44), com uma diferença de R$ 28,03 por unidade.

#### B. Braun

A B. Braun, empresa alemã fundada em 1839, é uma das líderes globais no setor de tecnologia médica, empregando mais de 63.000 pessoas em 64 países ^23^. Em 2023, a companhia registrou um volume de vendas superior a R$ 40 bilhões, abrangendo cerca de 5 mil produtos nas áreas de anestesia, medicina intensiva, cardiologia, hemodiálise e cirurgias ^23^. A empresa também investiu mais de R$ 2 bilhões em pesquisa e desenvolvimento no mesmo ano ^23^. Suas principais instalações de produção estão localizadas na União Europeia, Suíça, Estados Unidos, Malásia, Vietnã e Brasil, sendo que os dialisadores são fabricados na Alemanha ^23^. No Brasil, a B. Braun possui uma subsidiária, os Laboratórios B. Braun, desde a década de 1960, com sede no Rio de Janeiro e fábricas de bombas de infusão em São Gonçalo, empregando 1.339 pessoas ^23^. 

No Quadro 1 estão apresentadas as principais informações sobre as aquisições de dialisadores da B. Braun pela EBSERH. Constata-se que a participação da B. Braun no fornecimento de dialisadores para a EBSERH foi relativamente pequena no período analisado, especialmente considerando seu porte. A empresa forneceu poucos equipamentos a preços abaixo da média paga pela EBSERH, tornando-se a fabricante com menor contribuição para os custos de aquisição de dialisadores. Os hospitais da Região Nordeste foram os principais clientes dos produtos da B. Braun, que são distribuídos exclusivamente pelos Laboratórios B. Braun, em um modelo centralizado de vendas.

#### Fresenius Medical Care

A Fresenius Medical Care, originada da Farmácia Hirsch fundada em 1462 e adquirida pela família Fresenius em 1912, é uma líder global na oferta de produtos e serviços para pacientes com doença renal crônica, com mais de 4 mil clínicas de hemodiálise atendendo mais de 332 mil pacientes em todo o mundo ^24^. Em 2023, a empresa gerou receita superior a R$ 100 bilhões e realizou cerca de 52 milhões de sessões de hemodiálise ^24^. Com mais de quarenta plantas de produção em vinte países, a Fresenius fabrica máquinas de diálise, dialisadores e outros insumos, comercializando seus produtos em aproximadamente 150 países ^24^. A empresa é responsável por mais da metade das máquinas de hemodiálise utilizadas globalmente e investe pesadamente em pesquisa e desenvolvimento, com mais de R$ 700 milhões aplicados em 2019 e 1.157 pesquisadores dedicados ^24^. No Brasil, a Fresenius emprega cerca de 3 mil pessoas, com fábricas em Jaguariúna (São Paulo) e Campo Mourão (Paraná), além de 31 clínicas de hemodiálise e mais de 500 instalações clientes, operando mais de 18 mil máquinas no país ^24^. 

No Quadro 1 estão apresentadas as principais informações sobre as aquisições de dialisadores da Fresenius pela EBSERH. A Fresenius destacou-se pela diversidade de seus produtos, incluindo modelos específicos como o dialisador de alto fluxo FX PAED para uso pediátrico, o que possibilitou a empresa vender produtos com preços mais elevados, vencer mais licitações e atender a um número maior de clientes.

#### Gambro (Baxter)

Desde 2013, a Gambro faz parte do grupo Baxter, uma empresa estadunidense fundada em 1931 e inicialmente dedicada à fabricação de soluções intravenosas ^25^. Em 2023, as vendas globais da Baxter ultrapassaram R$ 70 bilhões, atendendo mais de 350 milhões de pacientes em mais de 100 países ^25^. A empresa emprega mais de 60 mil pessoas e possui sessenta fábricas ao redor do mundo, incluindo unidades nos Estados Unidos, onde são produzidos seus dialisadores ^25^. O portfólio da Baxter abrange diversas áreas da medicina, como nutrição parenteral, anestésicos, máquinas de hemodiálise e produtos para reconstrução de tecidos em cirurgias ^25^. No Brasil, a empresa iniciou suas operações em 1960, contando com mais de 1.200 funcionários distribuídos entre a fábrica, escritório, serviços técnicos e equipes de vendas, além de um armazém em Jundiaí (São Paulo) ^25^. No país, a Baxter fabrica soluções intravenosas, de diálise, bolsas e acessórios para infusão de medicamentos ^25^. 

No Quadro 1 estão apresentadas as principais informações sobre as aquisições de dialisadores da Gambro (Baxter) pela EBSERH. De acordo com os dados, a Gambro (Baxter) teve uma participação reduzida nas compras da EBSERH no período analisado, com o menor número de licitações vencidas e de modelos de dialisadores fornecidos. A empresa adota um sistema de distribuição próprio, centralizado, e suas vendas para a EBSERH ocorreram predominantemente na Região Nordeste.

#### Nipro

A Nipro, uma indústria japonesa fundada em 1954, iniciou suas atividades como distribuidora de vidrarias para a indústria farmacêutica ^26^. Atualmente, a empresa se concentra em três grandes áreas: produtos para saúde, farmacêutica e embalagens para produtos farmacêuticos, com centros de pesquisa dedicados a cada uma dessas áreas ^26^. Além do Japão, onde são produzidos seus dialisadores, a Nipro possui fábricas em países como Tailândia, China, Índia, Indonésia e Vietnã, empregando 4.388 pessoas ^26^. A empresa estabeleceu operações no Brasil em 1995, com plantas em Sorocaba (São Paulo) e Porto Alegre (Rio Grande do Sul), onde fabrica agulhas de fístula, cateteres intravenosos e produtos cardiopulmonares utilizados em cirurgias cardíacas ^26^. 

No Quadro 1 estão apresentadas as principais informações sobre as aquisições de dialisadores da Nipro pela EBSERH. De acordo com os dados, a Nipro obteve resultados significativos nas licitações da EBSERH, fornecendo mais de 14% do total de dialisadores adquiridos. A empresa conta com distribuidora própria, além de vender seus produtos para distribuidoras externas, o que lhe permite alcançar diversas regiões do Brasil.

#### Análise global das fabricantes

A partir da análise dos perfis dos fabricantes descritos anteriormente, percebe-se que todos os dialisadores adquiridos pela EBSERH no período analisado foram fabricados por empresas com sede em apenas cinco países: Alemanha (Fresenius Medical Care, B. Braun), China (Bain Medical), Estados Unidos da América (Baxter), Japão (Nipro) e Reino Unido (Allmed). O fato de certas cadeias de suprimento de dispositivos médicos dependerem de insumos geograficamente concentrados em alguns países já foi indicado em relatório da Organização para a Cooperação e Desenvolvimento Econômico (OCDE) ^27^. Esse tipo de concentração de produção está associado a uma série de riscos para o mercado, como a criação de gargalos de abastecimento, a vulnerabilidade geopolítica dos consumidores e o maior poder de negociação e controle de preços dos produtores. A própria OCDE defende que, para minimizar esses riscos, os países devem diversificar a origem desses produtos e, em determinados cenários, recomenda a intervenção dos governos para garantir sua capacidade de produção.

Além da concentração geográfica dessas empresas, é relevante observar que existem diferentes estratégias de mercado que podem ser adotadas para o sucesso comercial na produção de dialisadores. Enquanto a Allmed, a Bain Medical e a Nipro são empresas de porte menor, nas quais os dialisadores representam uma parte significativa do portfólio, a B. Braun, a Fresenius e a Baxter são grupos de maior dimensão, nos quais os dialisadores constituem apenas uma das diversas fontes de receita. Entre esses três grandes grupos, há abordagens estratégicas substancialmente distintas: a B. Braun e a Baxter adotam um catálogo horizontalizado de produtos, abrangendo várias áreas da medicina, enquanto a Fresenius optou por um catálogo verticalizado, especializando-se em todos os serviços e produtos necessários para pacientes com doenças renais. Na próxima seção, será apresentado um estudo detalhado sobre as principais características dos dialisadores fabricados por essas empresas e adquiridos pela EBSERH.

### Características dos dialisadores adquiridos pela EBSERH

#### Materiais e dimensões

Os principais materiais empregados na confecção das membranas de hemodiálise são as poliariletersulfonas, especialmente polissulfonas (PSF) e polietersulfonas (PES), conforme indicado no Quadro 2. Para modular a estrutura e a hidrofilicidade das membranas formadas, tendo em vista a aplicação na hemodiálise, é comum que outros polímeros sejam incorporados às poliariletersulfonas, como a poli(N-vinil-2-pirrolidona) e o polietilenoglicol ^28^ ^,^ ^29^. 

**Quadro 2:** Principais dimensões e materiais adotados pelos fabricantes de dialisadores adquiridos pela Empresa Brasileira de Serviços Hospitalares (EBSERH).

FABRICANTE	MODELO	MATERIAL	ESTERILIZAÇÃO	DIÂMETRO INTERNO (MICRÔMETROS)	ESPESSURA DE PAREDE (MICRÔMETROS)	PRESSÃO TRANSMEMBRANAR MÁXIMA (mmHG)
Allmed	H1	Polissulfona	Vapor	200	40	500
	H4	Polissulfona	Vapor	200	40	500
	PS100	Polissulfona	Vapor	200	40	500
	PS160	Polissulfona	Vapor	200	40	500
	PS180	Polissulfona	Vapor	200	40	500
B. Braun	PRO13H	Polissulfona	-	-	-	-
	PRO16L	Polissulfona	-	-	-	-
	PRO19H	Polissulfona	-	-	-	-
	PRO19L	Polissulfona	-	-	-	-
Fresenius	600S	Polissulfona	Vapor	220	35	600
	1000S	Polissulfona	Vapor	220	35	600
	F40S	Polissulfona	Vapor	220	35	600
	F50S	Polissulfona	Vapor	220	35	600
	F60S	Polissulfona	Vapor	220	35	600
	F70S	Polissulfona	Vapor	220	35	600
	HDF80S	Polissulfona	Vapor	220	35	600
	HDF100S	Polissulfona	Vapor	220	35	600
	EMIC2	Polissulfona	Vapor	220	35	600
	FX PAED	Polissulfona com polivinilpirrolidona	Vapor	220	35	600
Bain	B-14H	Polietersulfona	-	-	-	-
	B-16H	Polietersulfona	-	-	-	-
	B-18H	Polietersulfona	-	-	-	-
	B-20H	Polietersulfona	-	-	-	-
	B-20PF	Polietersulfona	-	-	-	-
Nipro	15H	Polietersulfona	Radiação Gama	200	40	500
	17H	Polietersulfona	Radiação Gama	200	40	500
	19H	Polietersulfona	Radiação Gama	200	40	500
	21H	Polietersulfona	Radiação Gama	200	40	500
	15L	Polietersulfona	Radiação Gama	200	40	500
	17L	Polietersulfona	Radiação Gama	200	40	500
	19L	Polietersulfona	Radiação Gama	200	40	500
	21L	Polietersulfona	Radiação Gama	200	40	500
Gambro	14L	Poliariletersulfona com polivinilpirrolidona	Vapor	215	50	600
	21L	Poliariletersulfona com polivinilpirrolidona	Vapor	215	50	600

**Figura 1:**
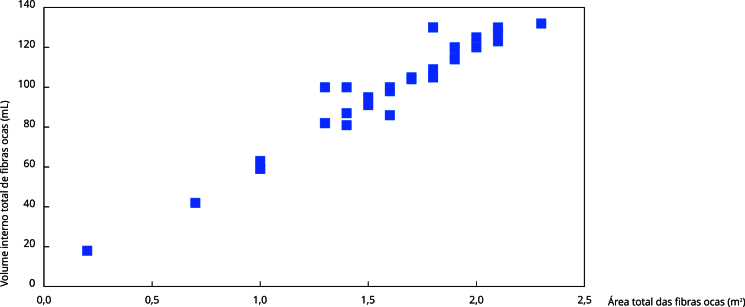
Volumes e áreas de fibras ocas para dialisadores comerciais.

Em relação às dimensões principais dos dialisadores adquiridos pela EBSERH, observa-se que os diâmetros das fibras variam entre 200 e 220 micrômetros, enquanto as espessuras das paredes dessas fibras estão entre 35 e 50 micrômetros. Apesar de muito finas, essas fibras devem suportar pressões transmembranares de até 600mmHg. Na Figura 1 é possível correlacionar o volume interno total das fibras ocas com a área total de filtração para os dialisadores listados no Quadro 2. Observa-se que são comercializados dialisadores que apresentam diferentes áreas e volumes de fibras ocas (*priming*), com variações entre 0,2 e 2,3m^2^ e 18 a 132mL, respectivamente.

Esses dados de diâmetro, área, espessura, volume e resistência à pressão de membranas comerciais podem ser úteis na regulação desses produtos, na elaboração de protocolos clínicos e na definição de critérios para compras governamentais. 

#### Propriedades de transporte das membranas de fibras ocas

**Figura 2:**
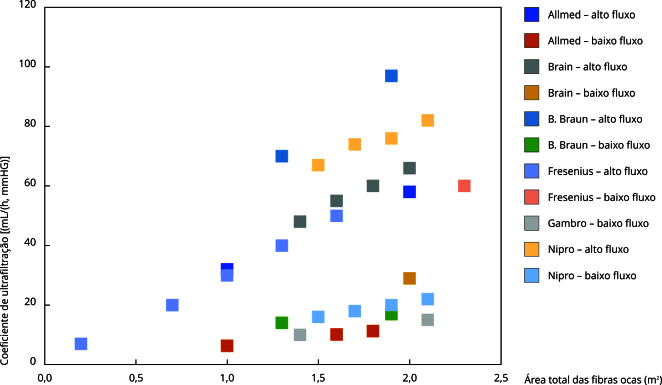
Coeficientes de ultrafiltração para dialisadores comerciais.

**Figura 3:**
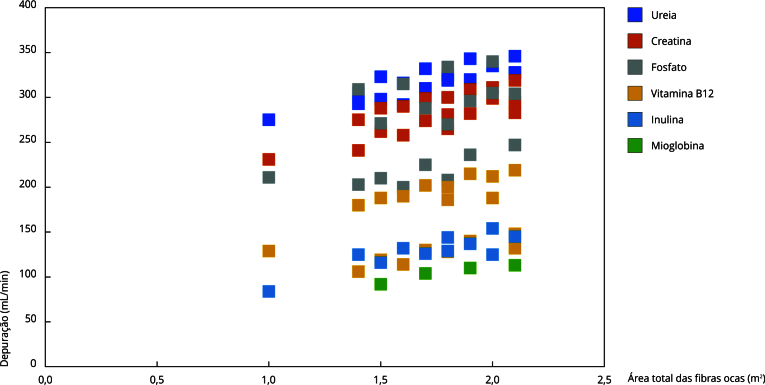
Dados de depuração para dialisadores comerciais. Medidas realizadas com fluxo de sangue de 400mL/min e fluxo de dialisato de 500mL/min.

As Figuras 2 e 3 ilustram, respectivamente, o coeficiente de ultrafiltração e os dados de depuração em função da área total das fibras ocas para os modelos de dialisadores listados no Quadro 2. Ao examinar a Figura 2, observa-se que os valores do coeficiente de ultrafiltração dos dialisadores comerciais seguem uma tendência linear esperada, com um aumento proporcional à área da membrana para cada categoria de dialisador. Ao examinar a Figura 3, observa-se que as moléculas mais pesadas, como a mioglobina (16.700Da), apresentam maior dificuldade para serem removidas do sangue, enquanto moléculas mais leves, como a ureia (60Da), são quase completamente eliminadas, o que confirma a tendência natural esperada para a remoção de solutos.

Esses dados de coeficiente de ultrafiltração e depuração de membranas comerciais podem ser úteis na avaliação do montante de investimento e do tipo de processo produtivo necessário para atingir o nível de qualidade exigido para competir com esses produtos importados. 

A apresentação destes aspectos técnicos descritivos a respeito dos dialisadores se justifica pelo fato de que o desenvolvimento de um parque industrial de membranas de hemodiálise envolveria competências em diversas áreas, como na síntese de polímeros, no preparo e caracterização de membranas, na montagem de módulos, na avaliação econômica de projetos e nos ensaios *in vitro* e *in vivo* para validar a metodologia e o produto obtidos. Sendo uma área de trabalho transdisciplinar e que requer a coordenação de profissionais em diferentes áreas, dados técnicos descritivos serão demandados em abundância durante as fases de implantação desse tipo de projeto. Dessa maneira, este trabalho poderá ser uma fonte de acesso para esses dados disponibilizados de forma clara e organizada.

### Política nacional para desenvolvimento de dialisadores

Os dados discutidos ao longo deste trabalho, especialmente as informações apresentadas no Quadro 1, demonstram a dependência tecnológica do Brasil de multinacionais estrangeiras para compra de dialisadores, um item de primeira necessidade para uma parcela significativa de sua população. Mais do que uma análise numérica de preços, prazos e tendências de mercado, esta pesquisa revela que nas últimas décadas o Brasil abdicou de ser protagonista no desenvolvimento de tecnologia própria neste segmento.

A dependência brasileira de dialisadores importados ilustra de forma precisa o mecanismo descrito na literatura sobre o Complexo Econômico-Industrial da Saúde (CEIS), segundo o qual a fragilidade da base produtiva nacional impede que a capacidade científica acumulada se converta em inovação efetiva ^30^. No caso específico desses dispositivos, a abertura comercial não acompanhada de uma política industrial ativa consolidou trajetórias de dependência tecnológica que se retroalimentam: a ausência de produção local reduz o aprendizado industrial, o que, por sua vez, reforça a necessidade de recorrer a fornecedores estrangeiros para suprir uma demanda essencial do SUS. 

Esse é mais um exemplo claro de que a política industrial não pode resumir-se em abertura econômica. É necessária a racionalização dos sistemas de proteção externa (tarifas, barreiras não-tarifárias, câmbio) e de promoção interna (incentivos, subsídios, financiamento), essenciais para a superação do atraso tecnológico e para a implantação de um novo segmento industrial ^31^. 

A indústria global de dispositivos médicos é marcada pela integração vertical de produtos e serviços, pelos investimentos massivos em pesquisa e desenvolvimento, pela forte intensidade tecnológica e pelos elevados níveis de concentração decorrentes de fusões e aquisições ^32^. Devido a todas essas características que muitas vezes são utilizadas como barreiras de entrada pelas líderes globais, o mercado de dispositivos médicos costuma ser de difícil acesso para novas empresas. Nesse contexto, o uso do poder de compra do Estado, especialmente via SUS, o incentivo à pesquisa e desenvolvimento, as políticas de financiamento e um marco regulatório adequado a essa realidade são instrumentos estratégicos que podem possibilitar o surgimento e a sustentação desses negócios. 

Especificamente tratando do segmento de dialisadores, o desenvolvimento da produção local pode transformar um setor dominado por importações em um componente importante no fortalecimento do CEIS brasileiro. Além de promover soberania sanitária e reduzir a vulnerabilidade geopolítica do país, uma iniciativa como essa gera inovações tecnológicas voltadas ao interesse público, cria massa crítica e ganho de experiência competitiva em segmentos de alta complexidade. Ademais, soluções adequadas à ampla realidade nacional e à lógica do sistema de saúde brasileiro podem atender a demandas comuns a um grande número de países de renda média e baixa, apresentando um elevado potencial de geração de parcerias ^33^.

Algumas circunstâncias como a vigência da Lei de Inovação e do Programa Nova Indústria Brasil ^11^ ^,^ ^12^ podem ser aproveitadas para iniciar esse movimento de reversão da dependência de importações. A estrutura para desenvolvimento de um parque fabril de membranas de hemodiálise deverá envolver a coordenação de diversos setores que possuem diferentes competências necessárias ao projeto (materiais, polímeros, membranas, biologia, medicina, enfermagem). Essas competências existem e estão disponíveis em território nacional, mas precisam ser articuladas adequadamente para alcance dos objetivos desejados.

Esse diagnóstico está de acordo com o contexto geral das políticas de inovação e industrialização brasileiras. Em trabalho recente, a Empresa Brasileira de Pesquisa e Inovação Industrial (EMBRAPII) publicou um estudo ^34^ em que, embora não cite especificamente a hemodiálise, defende a ampliação da capacidade de pesquisa e o incentivo à produção, priorizando produtos nacionais em processos de compras governamentais para atendimento ao SUS. Ainda avalia-se que investimentos e parcerias com instituições públicas e privadas são imprescindíveis para internalizar a transferência de tecnologia e nacionalizar produtos de alto valor agregado. Inserir os processos produtivos ligados à indústria de insumos para hemodiálise no radar dos decisores desses investimentos é fundamental para garantir a dignidade do tratamento dos pacientes brasileiros com doença renal crônica no longo prazo.

## Conclusão

Os resultados apresentados neste estudo forneceram uma visão do segmento de dialisadores no Brasil, com base em dados das aquisições realizadas pela EBSERH. Projeções realizadas a partir desses resultados precisam ser realizadas com cautela, considerando que a quantidade de dialisadores adquirida pela EBSERH correspondeu a apenas 1,8% do total de dialisadores importados pelo Brasil e que os processos analisados foram todos realizados no intervalo de um ano.

Foram explorados, na seção *Fabricantes dos Dialisadores Adquiridos pela EBSERH*, aspectos mercadológicos, incluindo os principais fabricantes, distribuidores, clientes, preços e estratégias competitivas, enquanto a seção *Características dos Dialisadores Adquiridos pela EBSERH* destacou os atributos físicos dos dialisadores, como materiais utilizados, dimensões e propriedades de transporte. Na seção *Política Nacional para Desenvolvimento de Dialisadores* foi feita uma síntese das questões abordadas nas seções anteriores, porém com enfoque na relação entre compras públicas e inovação em saúde. Essas análises serviram para aprofundar o entendimento do tema, identificar instituições relevantes e definir os limites tecnológicos, constituindo uma base de dados que poderá servir de insumo para definição de estratégias de desenvolvimento industrial no segmento de membranas de hemodiálise, principalmente no que diz respeito à análise de concorrentes e características de produtos de sucesso comercial.

Esta pesquisa tem potencial para contribuir para o desenvolvimento do segmento nacional de membranas de hemodiálise por evidenciar as lacunas na atuação do Brasil nesse setor. Futuros estudos que busquem analisar a viabilidade econômica e o impacto social da construção de indústrias de membranas de hemodiálise nacionais são necessários para o avanço do conhecimento na área. Além disso, pesquisas que se dediquem à construção de séries históricas de preços de dialisadores e insumos para hemodiálise em geral seriam de grande utilidade tanto para o mercado, quanto para o crescimento da base científica neste segmento. Recomenda-se ainda que trabalhos futuros aprofundem a análise realizada neste artigo por meio de modelagem estatística e comparações interinstitucionais.

## Data Availability

Os dados de pesquisa estão disponíveis mediante solicitação ao autor de correspondência.
